# Suggested explanations for the (in)effectiveness of nutrition information interventions among adults with a low socioeconomic status: a scoping review

**DOI:** 10.1017/jns.2022.42

**Published:** 2022-06-23

**Authors:** Tim van Meurs, Joost Oude Groeniger, Willem de Koster, Jeroen van der Waal

**Affiliations:** 1 Department of Public Administration and Sociology, Erasmus University Rotterdam, Burgemeester Oudlaan 50, 3062PA Rotterdam, the Netherlands; 2 Department of Public Health, Erasmus MC, Doctor Molewaterplein 40, 3015GD Rotterdam, the Netherlands

**Keywords:** Effectiveness, Health inequalities, Health information, Health literacy, Social inequalities

## Abstract

Unhealthy diets are a major threat to population health and are especially prevalent among those with a low socioeconomic status (SES). Health promotion initiatives often rely on nutrition information interventions (NIIs), but are usually less effective among adults with a low SES than in their high-SES counterparts. Explanations for this lower effectiveness are set out in extant studies. These have been conducted across a wide range of disciplines and subject fields and using a variety of methodological approaches. We have therefore conducted a scoping review to identify and synthesise the following: (1) explanations suggested in studies carried out in high-income countries for why NIIs are (in)effective among adults with a low SES and (2) whether these suggested explanations were studied empirically. Eight databases were searched for relevant studies published since 2009 across various disciplines. This identified 4951 papers, 27 of which were included in our review after screening. Only fifteen of these proposed an explanation for the (in)effectiveness of NIIs among adults with a low SES. The following four main themes were uncovered: health literacy, economic resources, social resources and convenience. Ten studies tested their explanations empirically, but the results were inconsistent. The reasons why NIIs are (in)effective among low-SES adults are therefore still largely unclear. Also, current literature predominantly relies on individualistic explanations, most notably focusing on psychological and economic attributes. Consequently, if the effectiveness of NIIs among low-SES populations is to be improved, future studies should examine a wider range of explanations and test them systematically and empirically.

## Introduction

Notwithstanding the substantial efforts being made by governmental, scientific and health institutions to promote good health, a significant difference remains in this regard between those in higher and lower socioeconomic status (SES) groups^(1)^. The persistence of this gap is partly the result of dietary inequalities^(2,3)^. These reflect the reality that health interventions aimed at improving what we eat are either ineffective among adults in the lower social strata, or less effective than among their higher-SES counterparts^(4,5)^. Consequently, nutritional interventions are often, and inadvertently, failing to narrow this SES health disparity. In particular, nutrition information interventions (NIIs) that encourage healthier eating by informing people ‘how to choose nutritious foods in order to follow guidelines for healthy eating’^(6)^ are frequently less successful at achieving their intended goals among those with a low SES^(7,8)^. Nevertheless, NIIs are still popular^(9)^, mainly because they are easy to execute and not particularly dependent on governmental decisions and the enactment of legislation.

It is, therefore, clear that the development of more effective and, specifically, more equitable NIIs requires an understanding of *why* current NIIs are (in)effective among low-SES groups. Nevertheless, there has been no comprehensive overview of the explanations suggested for why NIIs are (in)effective among these groups, nor of the extent to which these explanations have been studied empirically. Yet, since this reasoning probably differs across disciplines and research fields, it is important to synthesise this knowledge base. Consequently, we performed a scoping review to identify intervention studies conducted in high-income countries that examined the effectiveness of NIIs among low-SES groups (either specifically in these populations or that included a subgroup analysis). The review encompassed research conducted in a variety of fields, using various study designs, and with different types of NIIs. In particular, we carried out a thematic analysis to uncover the explanations suggested in the studies for the (in)effectiveness of these interventions among low-SES adults. We also examined whether these explanations were studied empirically. Our review was guided by the following research questions: (1) *What are the key explanations suggested in health intervention studies for why NIIs are (in)effective at improving health knowledge and achieving (intended) behavioural change among low-SES adults?* and (2) *Have these explanations been studied empirically?*

## Methods

We conducted a scoping review to answer our research questions^(10,11)^. Scoping reviews are commonly used to summarise, rather than evaluate, a particular field, which enabled us to ‘examine the extent, range and nature of research activity’^(10)^ relevant to the issue at hand. We conducted the review following the Preferred Reporting Items for Systematic Reviews and Meta-Analyses (PRISMA) checklist extension for scoping reviews^(12)^.

### Identifying relevant studies

The studies in our sample were obtained after a search of a variety of electronic databases, in particular by translating into a search syntax the concepts concerning (un)healthy diets that were closely related to the research questions. These were then combined with the terms ‘health information interventions’ and ‘socioeconomic status’, as well as their equivalent medical subject headings (MeSH). Abbreviations, synonyms and indicators were added to widen the search.

A variety of databases was used (Web of Science, Embase, Medline Ovid, Cochrane, Psyc INFO, Econ Lit, Abi/inform and Google Scholar) to ensure the inclusion of studies from diverse disciplines and research fields (Supplementary Appendix lists the search queries). Subsequently, references of the included studies were scanned to identify papers that may have been missed in the initial search, but none were detected.

### Study selection

All of the studies’ titles, abstracts and keywords, as well as the full texts, were screened independently by the first and last authors based on pre-set inclusion and exclusion criteria. Studies were included when they contained NIIs that: (1) were empirically discussed; (2) were produced by official institutions (e.g., governmental, scientific and health institutions); (3) had the aims of improving knowledge of health issues and/or changing (intended) behaviour; (4) concerned (un)healthy diets and (5) were examined on the basis of their effectiveness among low-SES groups. Studies were excluded when the intervention did not take place in a high-income Organisation for Economic Co-operation and Development (OECD) country or was not targeted exclusively at adults. The search period covered papers published from January 2009 to April 2019. The decision to use 2009 as a starting point was based on the landmark publication of the report ‘Closing the Gap in a Generation’. This was produced by the World Health Organisation's Commission on Social Determinants of Health^(13)^, and caused an upsurge in research focusing on reducing socioeconomic health inequalities.

### Charting data

Quotes were extracted from the papers concerning the studies’ designs, locations, outcome measures, intervention target groups, types of NII and identified effects on the low-SES participants. We also extracted details on the equitability of the intervention examined and the (suggested) explanations for why it was (in)effective. These quotes were then coded inductively by the first author.

### Collating, summarising and reporting findings

Coding was conducted based on a thematic analysis (the themes of the explanations proposed). Each study was assigned a theme (and potential additional themes) based on the reasons used to explain why the NII was (in)effective among the low-SES participants. These themes were then categorised using higher-level coding to facilitate the synthesis of the studies. Our analysis thus produced a scoping review of the different explanations currently suggested for the (in)effectiveness and their empirical value. To the best of our knowledge, this is the first review to focus on why NIIs are said to be (in)effective among adults with a low SES, rather than on whether they have an impact and, if so, to what extent.

## Results

### Descriptive numerical summary

**Fig. 1.**
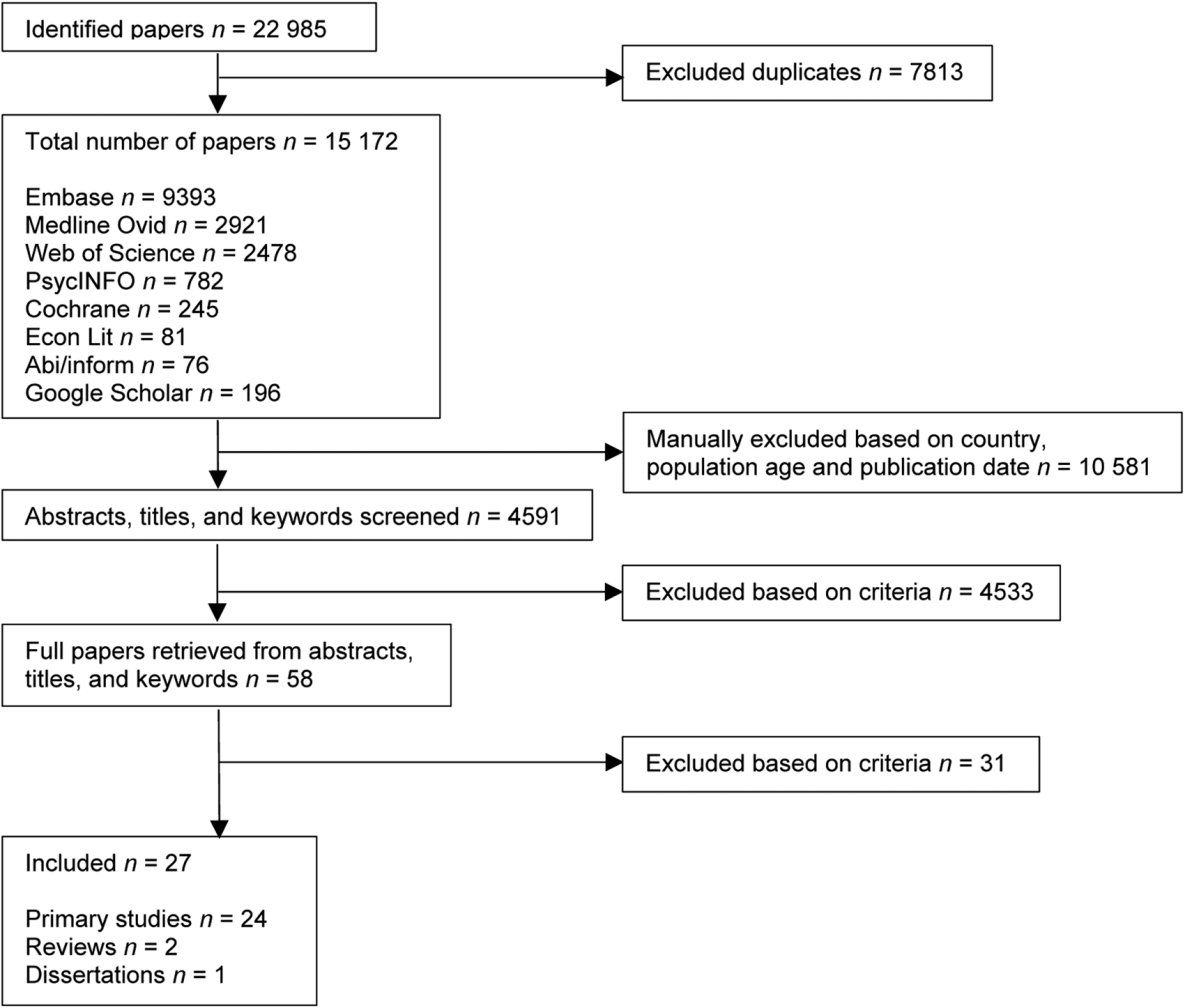
Flowchart of inclusions and exclusions.

The initial database search produced 22 985 entries, reduced to 15 172 after the removal of duplicates. Of these, 10 581 were pre-excluded based on the publication date (pre-2009), study population (not adults) and the country where the study was carried out (not a high-income OECD member). This reduced the sample to 4951 studies. Titles and abstracts were then reviewed, producing fifty-eight full texts for screening. This led to a sample comprising twenty-nine studies. Two of these were then excluded after careful consideration during the data-extraction phase, as they proved to be ineligible after all, leaving a final sample of twenty-seven studies for use in the thematic analysis. The inter-coder reliability for the full-text phase was 82⋅1 %. The coders subsequently reached a consensus by discussing whether to include or exclude the remaining studies that had initially been regarded as eligible by only one of them. Fig. 1 contains a detailed overview of the selection process.

Studies discussed nutritional or calorie-labelling (*n* 10); tailored (web-based) health information (*n* 6); general guidelines or recommendations (e.g., state-level guidelines, educational poster) (*n* 5); mass/multimedia campaigns (*n* 2); on-location information (e.g., point-of-purchase merchandising, grocery store interventions) (*n* 2); combined interventions with informational meetings (*n* 2); multicomponent education interventions (*n* 1) and online interventions (*n* 1). The NIIs examined in the studies were produced by the government (*n* 12); health institutions (*n* 7); science/academia (*n* 6) and non-governmental organisations (*n* 1). Two studies (both systematic literature reviews) did not specify the institution(s) that provided the health information.

Most of the studies concerned a single country, in particular: the United States (*n* 13); the Netherlands (*n* 5); New Zealand (*n* 2); the United Kingdom (*n* 2); Australia (*n* 1); France (*n* 1) and Norway (*n* 1). Two studies – both literature reviews – involved multiple countries. A cross-country comparison showed that the studies from the US had a disproportionately strong focus on (fast food menu) calorie-labelling, which was a feature in five of thirteen studies. The only other studies on labelling (one in the UK and one in Norway) focused more broadly on a variety of forms of labelling.

The crucial findings of the studies included in our thematic analysis are described in columns seven (effect among low SES) and eight (equity effect) in Table 1. Fifteen studies identified a positive intervention effect on their low-SES participants, five did not, and four had mixed results (a positive effect was found for some of the outcome measures). In three studies, the effect for low-SES participants was unclear. In terms of equity effects, only one study reported that the NII was completely equity-positive (i.e., inequalities decreased), whereas five identified negative equity effects, and seven highlighted similar effects across SES groups. Seven studies described mixed equity results, which generally meant that the equity findings differed per outcome. In one case^(28)^, these effects varied per SES indicator, while another was a systematic literature review that identified different equity effects in the studies included in its sample. Seven studies from the US were the only ones to focus exclusively on those with a low SES, with those conducted elsewhere merely examining entire populations. As a result, equity effects were not reported in over half of the US-based studies.

**Table 1.** Summary table of included studies

Ref.	Study location	Study design	Outcome measure(s)	Study population	Health information intervention	Effect among low SES?	Equity effect	Explanation theme	Empirically studied?
Backman *et al.* 2011^(14)^	USA	Quasi-experiment	Behavioural	Low SES	Point-of-purchase merchandising	Yes	N/A	No explanation	N/A
Blake *et al.* 2018^(15)^	Australia	Discrete choice experiment	Behavioural	Full pop.	Educational poster on sugar intake	Yes	+	Health literacy	In design
Blakely *et al.* 2011^(16)^	New Zealand	Randomised controlled trial (RCT)	Behavioural	Full pop.	Tailored nutrition education	No	0	No explanation	N/A
Breck *et al.* 2014^(17)^	USA	Cross-sectional	Behavioural	Full pop.	Calorie-labelling	Unclear	–	No explanation	N/A
Capacci and Mazzocchi 2011^(18)^	UK	Ex-post assessment;	Behavioural	Full pop.	Multimedia campaign	Yes	0	Economic resources	Suggested
Elbel *et al.* 2009^(19)^	USA	Cross-sectional	Behavioural	Low SES	Calorie-information	No	N/A	Health literacy	Suggested
Gans *et al.* 2009^(20)^	USA	RCT	Behavioural	Full pop.	Tailored information, video and print version	Yes	Mixed	Health literacy; personal appeal	In design
Gans *et al.* 2018^(21)^	USA	RCT	Behavioural	Low SES	Multicomponent educational intervention	Yes	N/A	No explanation	N/A
Gorton *et al.* 2009^(22)^	New Zealand	Cross-sectional	Knowledge	Full pop.	Nutritional label, four formats	Yes	0	Health literacy	In design
Hartmann-Boyce *et al.* 2018^(4)^	Multiple countries	SLR (of RCTs)	Behavioural	Full pop.	Grocery store interventions; labelling; educational messages	Mixed	Mixed	No explanation	N/A
Hersey *et al.* 2015^(23)^	USA	Pre–post-quasi-experimental design study	Behavioural	Low SES	Dietary guidelines; informational meetings	Yes	N/A	Social resources; economic resources	Suggested
Irz *et al.* 2015^(24)^	France	Calibration exercise (simulation)	Behavioural	Full pop.	Nutritional recommendations	Yes	Mixed	No explanation	N/A
Mancino and Kuchler 2012^(25)^	USA	Consumer demand modelling	Behavioural	Full pop.	Dietary guidelines	No	–	Economic resources; convenience; health literacy	Suggested
Masic *et al.* 2017^(26)^	UK	Forced choice questionnaire paradigm with independent groups	Behavioural	Full pop.	Nutritional label, four formats	Yes	0	Health literacy	In design
McGeary 2013^(27)^	USA	Cross-sectional	Behavioural	Full pop.	General state-level nutrition education	No	–	No explanation	N/A
Øvrum *et al.* 2012^(28)^	Norway	Choice experiment	Behavioural	Full pop.	Printed information; nutritional labels	Yes	Mixed	No explanation	N/A
Rameshbabu 2014^(29)^	USA	Randomised experimental design	Behavioural	Low SES	Information booklet; informational meetings	Yes	N/A	Self-regulatory skills	In design
Sarink *et al.* 2016^(5)^	Multiple countries	SLR	Behavioural; knowledge	Full pop.	Calorie-labelling	Mixed	–	Economic resources; social resources; health literacy	Suggested
Schindler *et al.* 2013^(30)^	USA	Focus groups	Behavioural	Low SES	Calorie-labelling	Unclear	N/A	Economic resources; convenience; health literacy	Tested
Springvloet *et al.* 2015^(31)^	The Netherlands	RCT	Behavioural	Full pop.	Web-based computer tailored nutritional education, two versions	Yes	Mixed	Health literacy	In design
Springvloet *et al.* 2015^(32)^	The Netherlands	RCT	Behavioural	Full pop.	Web-based computer tailored nutritional education, two versions	Yes	Mixed	Health literacy	In design
Springvloet *et al.* 2016^(33)^	The Netherlands	RCT	Behavioural	Full pop.	Web-based computer tailored nutritional education, two versions	Mixed	0	Health literacy	In design
Taksler and Elbel 2014^(34)^	USA	Difference-in-difference design; cross-sectional	Knowledge	Full pop.	Calorie-labelling	No	–	No explanation	N/A
Thunström 2019^(35)^	USA	Hypothetical experiment	Behavioural	Full pop.	Calorie-labelling	Unclear	0	No explanation	N/A
Verheijden *et al.* 2012^(36)^	The Netherlands	Cohort study	Behavioural	Full pop.	Mass media campaign, two waves	Mixed	Mixed	No explanation	N/A
Walsh *et al.* 2017^(37)^	USA	Non-randomised, quasi-experimental feasibility test	Behavioural	Low SES	Online educational modules; printed information	Yes	N/A	Health literacy	In design
Walthouwer *et al.* 2015^(38)^	The Netherlands	RCT	Behavioural	Full pop.	Web-based computer tailored nutritional education, text and video	Yes	0	No explanation	N/A

### Thematic analysis

The analysis uncovered four main themes, which were supplemented with a category given the name ‘other’. The subsections below are ordered according to the number of times a theme was identified, starting with the most common.

#### Health literacy

Twelve studies used the issue of ‘health literacy’ (i.e., being (un)able to understand the information contained in an NII) to explain the (in)effectiveness of the interventions among those with a low SES^(5,15,19,20,22,25,26,30–33,37)^. As an example, NIIs were described as succeeding in this group because they were ‘clear and simple’^(15)^ or had an approach that was ‘low literate [in] nature’^(20)^. In both cases, the NII was effective at reducing inequalities in nutritional health between the studies’ low- and high-SES participants. In other cases, too, simpler design elements were reported to be the reason for their effectiveness among those with a low SES^(22,26,37)^, although no increases in equity were identified.

Other studies argued that NIIs are less effective because low-SES groups find it harder to process the materials provided to them, reaching the conclusion that simpler interventions could improve equity effects^(5)^. The outcomes of the intervention tested by Springvloet *et al.*^(31–33)^ caused them to suggest that people with a low SES may be overwhelmed by the scope (in terms of content or quantity) of the NIIs provided to them. Meanwhile, the studies by Elbel *et al.*^(19)^ and Schindler *et al.*^(30)^, both of which examined the effectiveness of calorie-labelling, proposed that this kind of information is currently not clear enough and requires improvement if this type of NII is to succeed.

Mancino and Kuchler^(25)^ used a slightly different argument to highlight the importance of health literacy. In particular, they suggested that the dietary guidelines concerning wholegrain bread might have less of an impact on low-income consumers, since they may find it difficult to distinguish between wholegrain and non-wholegrain products (even if they understand the message that the former is the healthier option).

This theme was also the only one in which cross-country differences were observable. Although health literacy was discussed in the studies conducted in a number of countries (Australia, New Zealand, the Netherlands, the UK and the US), it was relatively less prominent in US-based studies: while health literacy was discussed in five of seven US-based studies that suggested an explanation for the (in)effectiveness of an NII, the concept mostly arose in combination with other themes. Conversely, save for a single UK-based study, non-US-based studies focused exclusively on health literacy as the explanation for the (in)effectiveness identified.

#### Economic resources

The second-most common theme was economic resources (*n* 5)^(5,18,23,25,30)^, which was given as a main reason for the limited effectiveness of NIIs among those in the low-SES group. Two studies^(23,25)^ suggested that the chief cause of this was the (un)affordability of healthy food. Indeed, even though the NII employed in the study by Hersey *et al.*^(23)^ did lead to an increase in the intake of fruit and vegetables among low-SES individuals, the daily amounts consumed did not accord with the NII's recommendations. The possible explanation suggested for this outcome was the high cost of the relevant products. Mancino and Kuchler^(25)^ echoed these findings, arguing that many in the low-SES group live in ‘areas with limited access to affordable and nutritious foods’, signalling the concept of ‘food deserts’. They also reported that finding inexpensive food was more important to their economically deprived participants than consuming healthy options. This was also highlighted as an issue by the interviewees in Schindler *et al.*'s study^(30)^.

Capacci and Mazzocchi^(18)^ likewise found that having limited economic resources impacted the effectiveness of their study's multimedia campaign among those with a low SES in their sample, making the argument that additional income support or raising the prices of unhealthy food could improve this. The final study to refer to this theme was conducted by Sarink *et al.*^(5)^, although it offered no further explanation beyond identifying deprivation as a potential factor.

#### Social resources

The third main theme identified was mentioned by two studies and focuses on the limited social resources of the low-SES group^(5,23)^. Hersey *et al.*^(23)^ suggested that social support might be relevant after noting that their participants discussed the information in the material provided to them with their peers. The authors thus proposed that such discussions could increase the backing for healthy food options, implying that NIIs might be less effective for those with a limited social support.

Meanwhile, Sarink *et al.*^(5)^ also suggest that there is greater uptake of the advice in NIIs if the intended target has more social resources. Although they do not elaborate on the underlying causal mechanisms, these authors do argue that adults with a low SES have relatively fewer such resources and, as a result, demonstrate only limited support for menu labelling.

#### Convenience

Two studies pointed to the convenience of unhealthy food as a potential reason for why the advice in NIIs is not adopted^(25,30)^. According to Mancino and Kuchler^(25)^, health was a lower priority than convenience for the lower-income consumers in their sample, who were unmoved by the intervention examined in the study. This was also an argument made by Schindler *et al.*^(30)^, who found that convenience was a factor in the decisions made about food options, even when a menu provided information on the calories in each dish. In particular, despite the recommendations in the NII employed in the research, their low-income participants continued to buy from fast food chains, stressing that they did so because the convenience of not having to buy and prepare food was more important to them than the issue of the price of fast food. The participants also pointed to a lack of time and, as a consequence, their disregard for information about calories. Nonetheless, neither of these studies identified why issues of convenience were more prevalent in their particular low-SES sample.

#### Others

Rameshbabu^(29)^ found that study participants who scored highly for self-regulation and self-efficacy were affected more by the NII being examined. She consequently argued that ‘teaching self-regulation skills serves to involve the individual in making the behaviour change rather than passively providing them with the information to do so’^(29)^. However, her study contained no comparisons of those in different SES groups.

Gans *et al.*^(20)^, meanwhile, noted that the NIIs used in their study, in part, worked better among the low-SES respondents, because the material was ‘more personally relevant’: these interventions addressed the participants directly by using their name and tailoring the health information message to them as individuals, contributing to them being ‘more positive about how interesting and personally relevant’ this tailored information was. Their study was the only one to mention the tailored nature of NIIs as a possible reason for their effectiveness among low-SES groups, even though other studies have also examined these effects. However, these other studies do not specifically suggest that the tailoring of such interventions – and their resulting personal appeal – is an explanation for the extent of their effectiveness in these individuals. As a consequence, we have not included them within this theme.

#### No explanation

A key finding of our review was that twelve of the twenty-seven studies analysed did not include any explanation at all for why NIIs are (in)effective among low-SES groups^(4,14,16,17,21,24,27,28,34–36,38)^; instead, these studies simply reported their results, with no discussion of possible reasons for these outcomes.

### Empirical scrutiny of the suggested explanations

Of the twenty-seven studies included in the analysis, ten contained some empirical scrutiny of the explanations suggested for the (in)effectiveness of an NII among their low-SES participants. The most direct evidence came from focus group interviews^(30)^, which discussed various factors in order to identify why calorie information in fast food restaurants is rarely considered. The issues considered above all others by the participants were, most notably, clarity (i.e., health literacy) and convenience.

Nine studies referred to the design of an NII to account for its (in)effectiveness. Two tested different types of nutritional labels^(22,26)^ finding that simplified versions were more effective among low-SES groups. These included traffic-light labelling (using colours to indicate the healthiness of an item) and physical-activity labels (indicating the amount of exercise required to burn off the calories in the product). These approaches thus reduce the need for health literacy to ensure the effectiveness of an intervention. This is in contrast to numerical labels, which simply state the nutritional content in absolute numbers or as a percentage of daily intake. It should be noted that this simplified form of nutritional information also resonated with those in high-SES groups and, as a result, did not lead to any equity changes.

Six studies made the claim that the analysed NII had been developed with the specific goal that the information should be comprehensible to those with lower health literacy^(15,20,32,33,37,39)^, implying that any disparity in relation to this factor is the reason for the relative ineffectiveness of NIIs among low-SES groups. Nonetheless, the outcomes of these studies are inconsistent, with some producing equity-positive results and others equity-neutral or equity-negative outcomes. Moreover, none of the studies based on NIIs that were easy to understand were compared to versions containing less digestible information.

As well as taking health literacy into account in the design of their study, the tailored nature of the NII examined by Gans *et al.*^(20)^ was also claimed to be a reason for its effectivity, as it was deemed to be more personally appealing to those with a low SES. It should be noted, however, that an equity effect was only identified for one of the four outcome measures (change in the intake of fruit and vegetables at 7 months follow-up); for the other three (change in the fruit and vegetable intake at 4 months, and in the intake of fats at 4 and 7 months) no such effects were uncovered. Nevertheless, this combination of equity-neutral and equity-positive results does suggest that the use of tailored nutrition information takes us a step closer to reducing inequality. This seems to be endorsed in the studies by Springvloet *et al.*^(32,33,39)^ and Walthouwer *et al.*^(38)^, whose mainly equity-neutral or equity-positive results were achieved with tailored NIIs. Nonetheless, it should be noted that neither study attributes the effectiveness of the interventions examined to their tailored nature.

Finally, Rameshbabu^(29)^ found that promoting self-regulatory skills within an informational message had a positive impact on the extent to which the material was absorbed and acted upon. While the intervention alone also seemed to influence the study's respondents positively, adding information about self-regulation increased this effect significantly. Nonetheless, with participants exclusively from low-SES groups (non-academic employees at one university), it was not possible for the study to make claims about equitability, i.e., it was unable to determine whether the endorsement of self-regulatory skills alongside an informational message would affect high-SES groups to a different extent.

## Conclusion and discussion

Our scoping review identified twenty-seven studies that examined the effectiveness of NIIs among adults with a low SES. While most of the interventions investigated were shown to be effective among low-SES groups, they were often just as, or more, effective among those whose SES was high. Our thematic analysis revealed that almost half of the twenty-seven studies offered no explanation for the (lack of) impact on low-SES groups. In those that did, four main themes were identified: health literacy, economic resources, social resources and convenience; there were two further explanations that did not fit within these themes: self-regulation/self-efficacy and personal appeal.

Ten of the studies examined included some form of empirical research on the tenability of the explanations proposed. These predominately targeted the issue of ‘health literacy’ and the provision of simplified and easily digestible information. However, since these studies had inconsistent outcomes (equity-positive, equity-neutral and equity-negative) and most did not include a clear comparison group (e.g., information not adjusted to the level of literacy), it was not possible to determine the empirical tenability of this explanation. This suggests that an intervention that is only easy to understand is no panacea when it comes to reducing the nutritional health inequalities that exist today.

The most direct empirical evidence came from focus group interviews. Nonetheless, the qualitative nature of this type of study does not enable findings to be generalised to the population at large. Moreover, the participants were exclusively from low-SES groups, with most also having an ethnic minority background. This makes it difficult to determine whether the attitudes and actions reported were the results of a low SES, a particular ethnic background or a combination thereof.

Overall, therefore, it is still unclear why the equity effects of NIIs are inconsistent. In large part, this is because many of the studies examined were unable to test for differential effects (e.g., they were not powered to test the moderating effects of SES). However, even those that did include such an analysis did not always offer an explanation of their findings, perhaps because no between-group differences were identified. Nevertheless, even these limited interventions may provide valuable insights, as it is clearly more common for NIIs to be less effective among those in lower-SES groups. Accordingly, if the impact of such information is to be improved, it is important for intervention studies to focus more on *why* – instead of just on *whether* – some NIIs are effective and equitable and others are not.

### Implications

This is the first review to focus on *why* NIIs are (in)effective among those in low-SES groups. Our findings emphasise the need to add an explanatory perspective to a field that primarily focuses on impact assessments. Using research designs that enable determining why an NII is (in)effective in specific target groups could, however, provide the crucial information required to develop more effective – and more equitable – interventions. Moreover, to achieve a better understanding of the mechanisms that explain why an NII is more or less impactful among lower-SES groups, it is crucial that valid arguments are provided about why a specific approach would affect the impact of an intervention *and* why this mechanism may be distributed differentially between socioeconomic groups. Each pathway determines the effectiveness of an NII and should, as a result, be detailed enough to enable the design of more effective campaigns. Moreover, this approach should, perhaps, not only be limited to NIIs, since any health promotion intervention would benefit from its creators knowing why and how it is likely to be (in)effective, both generally and per SES group.

Our scoping review revealed a dearth of studies that conducted a rigorous, empirical examination of whether the explanations could actually account for the effects observed. This means it is impossible to make any empirically substantiated claims about why NIIs are (in)effective among low-SES groups. Nevertheless, our review has identified the *types* of explanation proposed, most notably health literacy and economic resources. If these explanations do, in fact, have an empirical basis, NIIs could become more equitable by relying less on the information-processing capacity of the intended recipient, and more on increasing the affordability and availability of the touted healthy food products. Removing any cognitive and financial barriers should then lead to the creation of interventions that are more successful among lower-SES groups.

Finally, it is important to note that the explanations suggested in the studies we examined are predominantly individualistic accounts, most notably focusing on psychological and economic attributes. As such, the possible relevance of sociocultural conditions in shaping the uptake of health knowledge has not yet been covered systematically. The field may, therefore, benefit from adding non-individualistic explanations, e.g., from fields like sociology and anthropology^(40–42)^.

### Limitations

This research has some limitations. First, only papers printed in English were considered for inclusion, meaning that a considerable number of studies written in other languages were excluded. The inclusion of more languages may have given a more complete picture of the field. Moreover, various studies in our thematic analysis focused on actual behavioural change rather than information uptake. This could have led to the relatively high number of times that economic factors were suggested as the reason for the (in)effectiveness of the interventions investigated: acting on information probably depends more on economic resources than is the case for its uptake. Furthermore, the relationship between information uptake and behavioural change has been contested^(43)^, which may partly explain the relatively low impact of the analysed interventions on behavioural change.

## Conclusion

Our scoping review has highlighted that only about half of the studies to examine NIIs suggest any explanations for their (in)effectiveness among those with a low SES; focusing mainly on cognitive and financial factors. Moreover, only about a third of these studies investigated empirically whether those explanations did actually account for the (in)effectiveness identified. This makes it difficult to learn lessons from past interventions. Future intervention studies should therefore focus more on establishing empirically why NIIs do, or do not, work as intended. This information is essential if we are to confront and reduce the considerable health inequalities that persist across the globe.

## Supplementary material

For supplementary material accompanying this paper visit https://doi.org/10.1017/jns.2022.42. click here to view supplementary material
