# Supplementary material for: Suggested explanations for the (in)effectiveness of nutrition information interventions among adults with a low socioeconomic status: a scoping review
Source: J Nutr Sci. 2022 Jun 23;11:e50. doi: 10.1017/jns.2022.42 (PMC9241061; doi:10.1017/jns.2022.42)
Supplement: Supplementary file 1 [file S2048679022000428sup001.docx]

Appendix A – Search terms used for each database

**Embase – Unhealthy diet – 8527 refs**

(diet/exp OR 'dietary intake'/de OR 'food intake'/de OR 'caloric density'/exp OR 'caloric intake'/exp OR 'carbohydrate intake'/exp OR 'diet restriction'/exp OR 'dietary reference intake'/exp OR 'fat intake'/exp OR 'maternal nutrition'/exp OR 'nutrition'/de OR 'feeding behavior'/de OR 'eating habit'/de OR 'food preference'/de OR 'soft drink'/de OR 'sweetened beverage'/exp OR (diet OR dieting OR diets OR dietar* OR ((soft OR sweet*) NEAR/3 (drink* OR beverage*)) OR ((feed* OR eat* OR food* OR diet*) NEAR/3 (behavior* OR behaviour* OR habit* OR preferenc* OR pattern*)) OR ((nutrition OR food* OR beverage* OR nutrient* OR micronutrient* OR macronutrient* OR vitamin* OR calor* OR energ* OR vegetable* OR fruit* OR fat OR fats OR fatty OR sugar* OR carbohydrate* OR glucose* OR sodium* OR salt* OR cholesterol*) NEAR/6 (intake* OR ingest*  OR supplement* OR consum* OR restrict* OR depriv*)) OR ((weight*) NEAR/3 (manag*)) OR ((well OR under) NEXT/1 nourish*) OR ((well OR under) NEXT/1 nourish*)):ab,ti) **AND** (policy/exp OR politics/de OR government/exp  OR 'legal aspect'/de OR deregulation/exp OR 'government regulation'/exp OR law/exp OR 'law enforcement'/exp OR 'mandatory program'/exp OR 'mass communication'/de OR 'mass medium'/de OR 'consumer health information'/de OR 'information dissemination'/de OR 'information literacy'/de OR 'information seeking'/de OR (regulation* OR government* OR law OR laws OR policy OR policies OR ((weight*) NEAR/3 (manag*)) OR ((information*) NEAR/3 (consumer-health* OR disseminat* OR literac* OR seek*)) OR ((diet OR nutrition OR sugar* OR fat OR fats*) NEAR/3 (restrict* OR act OR acts OR price OR pricing OR tax OR taxes OR taxation)) OR legal* OR illegal* OR hotline* OR quitline* OR (mass NEXT/1 (media OR medium OR communicat*)) OR ordinanc* OR prohibit* OR decree* OR enactment* OR mandator* OR campaign* OR ((pictorial OR graphic) NEAR/3 (warning*)) OR packaging OR mpower OR advertizing OR advertising OR legislat* OR statut* OR (('population-based' OR 'public health') NEAR/3 strateg*)):ab,ti) **AND** ('socioeconomics'/exp OR 'social status'/exp OR (((digital*) NEAR/3 (devide* OR inequalit*)) OR socioeconomic* OR socio-economic* OR ((economic* OR educat* OR career* OR job OR jobs OR work OR profession* OR social* OR socio OR sociocultur* OR socio-cultur* OR sociodemograph* OR socio-demograph* OR occupat* OR employ*) NEAR/3 (status* OR achievement* OR condition* OR rank* OR rank* OR standing* OR state OR background* OR class* OR mobilit* OR deprivat* OR disadvantag* OR equalt* OR inequalt* OR low OR lower)) OR literac* OR illitera* OR ((abilit* OR capabilit* OR skill*) NEAR/3 (read* OR learn*)) OR ((living) NEXT/1 (standard*)) OR poverty OR income* OR employed OR unemployed OR remunerat* OR salary OR salaries):ab,ti) *NOT ([animals]/lim NOT [humans]/lim) AND [english]/lim NOT ([Conference Abstract]/lim AND [1800-2016]/py)*

**Medline – 7761 refs**

(exp Diet/ OR Eating/ OR Energy Intake/ OR exp Maternal Nutritional Physiological Phenomena/ OR Nutritional Status/ OR Feeding Behavior/ OR Food Preferences/ OR soft drink/ OR (diet OR dieting OR diets OR dietar* OR ((soft OR sweet*) ADJ3 (drink* OR beverage*)) OR ((feed* OR eat* OR food* OR diet*) ADJ3 (behavior* OR behaviour* OR habit* OR preferenc* OR pattern*)) OR ((nutrition OR food* OR beverage* OR nutrient* OR micronutrient* OR macronutrient* OR vitamin* OR calor* OR energ* OR vegetable* OR fruit* OR fat OR fats OR fatty OR sugar* OR carbohydrate* OR glucose* OR sodium* OR salt* OR cholesterol*) ADJ6 (intake* OR ingest*  OR supplement* OR consum* OR restrict* OR depriv*)) OR ((weight*) ADJ3 (manag*)) OR ((well OR under) ADJ nourish*) OR ((well OR under) ADJ nourish*)).ab,ti.) **AND** (exp Policy/ OR Politics/ OR exp Government/ OR exp Jurisprudence/ OR Government Regulation/  OR exp Legislation, Drug/ OR Antitrust Laws/ OR Law Enforcement/ OR exp Mandatory Programs/ OR exp Mass Media/ OR exp Consumer Health Information/ OR Information Dissemination/ OR exp Information Literacy/ OR Information Seeking Behavior/ OR (regulation* OR government* OR law OR laws OR policy OR policies OR ((weight*) ADJ3 (manag*)) OR ((information*) ADJ3 (consumer-health* OR disseminat* OR literac* OR seek*)) OR ((diet OR nutrition OR sugar* OR fat OR fats*) ADJ3 (restrict* OR act OR acts OR price OR pricing OR tax OR taxes OR taxation)) OR legal* OR illegal* OR hotline* OR quitline* OR (mass ADJ (media OR medium OR communicat*)) OR ordinanc* OR prohibit* OR decree* OR enactment* OR mandator* OR campaign* OR ((pictorial OR graphic) ADJ3 (warning*)) OR packaging OR mpower OR advertizing OR advertising OR legislat* OR statut* OR ((population-based OR public health) ADJ3 strateg*)).ab,ti.) **AND** (exp Socioeconomic Factors/ OR exp Social Class/ OR (((digital*) ADJ3 (devide* OR inequalit*)) OR socioeconomic* OR socio-economic* OR ((economic* OR educat* OR career* OR job OR jobs OR work OR profession* OR social* OR socio OR sociocultur* OR socio-cultur* OR sociodemograph* OR socio-demograph* OR occupat* OR employ*) ADJ3 (status* OR achievement* OR condition* OR rank* OR rank* OR standing* OR state OR background* OR class* OR mobilit* OR deprivat* OR disadvantag* OR equalt* OR inequalt* OR low OR lower)) OR literac* OR illitera* OR ((abilit* OR capabilit* OR skill*) ADJ3 (read* OR learn*)) OR ((living) ADJ (standard*)) OR poverty OR income* OR employed OR unemployed OR remunerat* OR salary OR salaries).ab,ti.) *NOT (exp animals/ NOT humans/) AND english.la.*

**Cochrane (RCTs) – 570 refs**

((diet OR dieting OR diets OR dietar* OR ((soft OR sweet*) NEAR/3 (drink* OR beverage*)) OR ((feed* OR eat* OR food* OR diet*) NEAR/3 (behavior* OR behaviour* OR habit* OR preferenc* OR pattern*)) OR ((nutrition OR food* OR beverage* OR nutrient* OR micronutrient* OR macronutrient* OR vitamin* OR calor* OR energ* OR vegetable* OR fruit* OR fat OR fats OR fatty OR sugar* OR carbohydrate* OR glucose* OR sodium* OR salt* OR cholesterol*) NEAR/6 (intake* OR ingest*  OR supplement* OR consum* OR restrict* OR depriv*)) OR ((weight*) NEAR/3 (manag*)) OR ((well OR under) NEXT/1 nourish*) OR ((well OR under) NEXT/1 nourish*)):ab,ti) **AND** ((regulation* OR government* OR law OR laws OR policy OR policies OR ((weight*) NEAR/3 (manag*)) OR ((information*) NEAR/3 ((consumer NEXT/1 health*) OR disseminat* OR literac* OR seek*)) OR ((diet OR nutrition OR sugar* OR fat OR fats*) NEAR/3 (restrict* OR act OR acts OR price OR pricing OR tax OR taxes OR taxation)) OR legal* OR illegal* OR hotline* OR quitline* OR (mass NEXT/1 (media OR medium OR communicat*)) OR ordinanc* OR prohibit* OR decree* OR enactment* OR mandator* OR campaign* OR ((pictorial OR graphic) NEAR/3 (warning*)) OR packaging OR mpower OR advertizing OR advertising OR legislat* OR statut* OR (('population-based' OR 'public health') NEAR/3 strateg*)):ab,ti) **AND** ((((digital*) NEAR/3 (devide* OR inequalit*)) OR socioeconomic* OR (socio NEXT/1 economic*) OR ((economic* OR educat* OR career* OR job OR jobs OR work OR profession* OR social* OR socio OR sociocultur* OR (socio NEXT/1 cultur*) OR sociodemograph* OR (socio NEXT/1 demograph*) OR occupat* OR employ*) NEAR/3 (status* OR achievement* OR condition* OR rank* OR rank* OR standing* OR state OR background* OR class* OR mobilit* OR deprivat* OR disadvantag* OR equalt* OR inequalt* OR low OR lower)) OR literac* OR illitera* OR ((abilit* OR capabilit* OR skill*) NEAR/3 (read* OR learn*)) OR ((living) NEXT/1 (standard*)) OR poverty OR income* OR employed OR unemployed OR remunerat* OR salary OR salaries):ab,ti)

**Web of Science – 8643 refs**

TS=(((diet OR dieting OR diets OR dietar* OR ((soft OR sweet*) NEAR/2 (drink* OR beverage*)) OR ((feed* OR eat* OR food* OR diet*) NEAR/2 (behavior* OR behaviour* OR habit* OR preferenc* OR pattern*)) OR ((nutrition OR food* OR beverage* OR nutrient* OR micronutrient* OR macronutrient* OR vitamin* OR calor* OR energ* OR vegetable* OR fruit* OR fat OR fats OR fatty OR sugar* OR carbohydrate* OR glucose* OR sodium* OR salt* OR cholesterol*) NEAR/5 (intake* OR ingest*  OR supplement* OR consum* OR restrict* OR depriv*)) OR ((weight*) NEAR/2 (manag*)) OR ((well OR under) NEAR/1 nourish*) OR ((well OR under) NEAR/1 nourish*))) **AND** ((regulation* OR government* OR law OR laws OR policy OR policies OR ((weight*) NEAR/2 (manag*)) OR ((information*) NEAR/2 (consumer-health* OR disseminat* OR literac* OR seek*)) OR ((diet OR nutrition OR sugar* OR fat OR fats*) NEAR/2 (restrict* OR act OR acts OR price OR pricing OR tax OR taxes OR taxation)) OR legal* OR illegal* OR hotline* OR quitline* OR (mass NEAR/1 (media OR medium OR communicat*)) OR ordinanc* OR prohibit* OR decree* OR enactment* OR mandator* OR campaign* OR ((pictorial OR graphic) NEAR/2 (warning*)) OR packaging OR mpower OR advertizing OR advertising OR legislat* OR statut* OR (("population-based" OR "public health") NEAR/2 strateg*))) **AND** ((((digital*) NEAR/2 (devide* OR inequalit*)) OR socioeconomic* OR socio-economic* OR ((economic* OR educat* OR career* OR job OR jobs OR work OR profession* OR social* OR socio OR sociocultur* OR socio-cultur* OR sociodemograph* OR socio-demograph* OR occupat* OR employ*) NEAR/2 (status* OR achievement* OR condition* OR rank* OR rank* OR standing* OR state OR background* OR class* OR mobilit* OR deprivat* OR disadvantag* OR equalt* OR inequalt* OR low OR lower)) OR literac* OR illitera* OR ((abilit* OR capabilit* OR skill*) NEAR/2 (read* OR learn*)) OR ((living) NEAR/1 (standard*)) OR poverty OR income* OR employed OR unemployed OR remunerat* OR salary OR salaries)) NOT ((animal* OR rat OR rats OR mouse OR mice OR murine OR dog OR dogs OR canine OR cat OR cats OR feline OR rabbit OR cow OR cows OR bovine OR rodent* OR sheep OR ovine OR pig OR swine OR porcine OR veterinar* OR chick* OR zebrafish* OR baboon* OR nonhuman* OR primate* OR cattle* OR goose OR geese OR duck OR macaque* OR avian* OR bird* OR fish*) NOT (human* OR patient* OR women OR woman OR men OR man))) AND DT=(Article OR Review) AND LA=(English)

**PsycINFO – 1619 refs**

(exp Diets/ OR Eating Behavior/ OR Food Intake/ OR exp Nutrition/ OR Food Preferences/ OR (diet OR dieting OR diets OR dietar* OR ((soft OR sweet*) ADJ3 (drink* OR beverage*)) OR ((feed* OR eat* OR food* OR diet*) ADJ3 (behavior* OR behaviour* OR habit* OR preferenc* OR pattern*)) OR ((nutrition OR food* OR beverage* OR nutrient* OR micronutrient* OR macronutrient* OR vitamin* OR calor* OR energ* OR vegetable* OR fruit* OR fat OR fats OR fatty OR sugar* OR carbohydrate* OR glucose* OR sodium* OR salt* OR cholesterol*) ADJ6 (intake* OR ingest*  OR supplement* OR consum* OR restrict* OR depriv*)) OR ((weight*) ADJ3 (manag*)) OR ((well OR under) ADJ nourish*) OR ((well OR under) ADJ nourish*)).ab,ti.) **AND** (exp Health Care Policy/ OR Politics/ OR exp Government/ OR exp "Law (Government)"/ OR Government Policy Making/  OR exp Drug Laws/ OR Law Enforcement/ OR exp Mass Media/ OR Information Dissemination/ OR exp Information Literacy/ OR Information Seeking/ OR (regulation* OR government* OR law OR laws OR policy OR policies OR ((weight*) ADJ3 (manag*)) OR ((information*) ADJ3 (consumer-health* OR disseminat* OR literac* OR seek*)) OR ((diet OR nutrition OR sugar* OR fat OR fats*) ADJ3 (restrict* OR act OR acts OR price OR pricing OR tax OR taxes OR taxation)) OR legal* OR illegal* OR hotline* OR quitline* OR (mass ADJ (media OR medium OR communicat*)) OR ordinanc* OR prohibit* OR decree* OR enactment* OR mandator* OR campaign* OR ((pictorial OR graphic) ADJ3 (warning*)) OR packaging OR mpower OR advertizing OR advertising OR legislat* OR statut* OR ((population-based OR public health) ADJ3 strateg*)).ab,ti.) **AND** (exp Socioeconomic Status/ OR exp Socioeconomic Class Attitudes/ OR (((digital*) ADJ3 (devide* OR inequalit*)) OR socioeconomic* OR socio-economic* OR ((economic* OR educat* OR career* OR job OR jobs OR work OR profession* OR social* OR socio OR sociocultur* OR socio-cultur* OR sociodemograph* OR socio-demograph* OR occupat* OR employ*) ADJ3 (status* OR achievement* OR condition* OR rank* OR rank* OR standing* OR state OR background* OR class* OR mobilit* OR deprivat* OR disadvantag* OR equalt* OR inequalt* OR low OR lower)) OR literac* OR illitera* OR ((abilit* OR capabilit* OR skill*) ADJ3 (read* OR learn*)) OR ((living) ADJ (standard*)) OR poverty OR income* OR employed OR unemployed OR remunerat* OR salary OR salaries).ab,ti.) *NOT (exp animals/ NOT humans/) AND english.la.*

**Econ Lit – 118 refs**

AB,TI((((diet OR dieting OR diets OR dietar* OR ((soft OR sweet*) N2 (drink* OR beverage*)) OR ((feed* OR eat* OR food* OR diet*) N2 (behavior* OR behaviour* OR habit* OR preferenc* OR pattern*)) OR ((nutrition OR food* OR beverage* OR nutrient* OR micronutrient* OR macronutrient* OR vitamin* OR calor* OR energ* OR vegetable* OR fruit* OR fat OR fats OR fatty OR sugar* OR carbohydrate* OR glucose* OR sodium* OR salt* OR cholesterol*) N5 (intake* OR ingest*  OR supplement* OR consum* OR restrict* OR depriv*)) OR ((weight*) N2 (manag*)) OR ((well OR under) N1 nourish*) OR ((well OR under) N1 nourish*)))) **AND** (((regulation* OR government* OR law OR laws OR policy OR policies OR ((weight*) N2 (manag*)) OR ((information*) N2 (consumer-health* OR disseminat* OR literac* OR seek*)) OR ((diet OR nutrition OR sugar* OR fat OR fats*) N2 (restrict* OR act OR acts OR price OR pricing OR tax OR taxes OR taxation)) OR legal* OR illegal* OR hotline* OR quitline* OR (mass N1 (media OR medium OR communicat*)) OR ordinanc* OR prohibit* OR decree* OR enactment* OR mandator* OR campaign* OR ((pictorial OR graphic) N2 (warning*)) OR packaging OR mpower OR advertizing OR advertising OR legislat* OR statut* OR ((population-based OR public health) N2 strateg*)))) **AND** (((((digital*) N2 (devide* OR inequalit*)) OR socioeconomic* OR socio-economic* OR ((economic* OR educat* OR career* OR job OR jobs OR work OR profession* OR social* OR socio OR sociocultur* OR socio-cultur* OR sociodemograph* OR socio-demograph* OR occupat* OR employ*) N2 (status* OR achievement* OR condition* OR rank* OR rank* OR standing* OR state OR background* OR class* OR mobilit* OR deprivat* OR disadvantag* OR equalt* OR inequalt* OR low OR lower)) OR literac* OR illitera* OR ((abilit* OR capabilit* OR skill*) N2 (read* OR learn*)) OR ((living) N1 (standard*)) OR poverty OR income* OR employed OR unemployed OR remunerat* OR salary OR salaries))))

**Abi/inform 190 refs**

AB,TI((((diet OR dieting OR diets OR dietar* OR ((soft OR sweet*) N2 (drink* OR beverage*)) OR ((feed* OR eat* OR food* OR diet*) N2 (behavior* OR behaviour* OR habit* OR preferenc* OR pattern*)) OR ((nutrition OR food* OR beverage* OR nutrient* OR micronutrient* OR macronutrient* OR vitamin* OR calor* OR energ* OR vegetable* OR fruit* OR fat OR fats OR fatty OR sugar* OR carbohydrate* OR glucose* OR sodium* OR salt* OR cholesterol*) N5 (intake* OR ingest*  OR supplement* OR consum* OR restrict* OR depriv*)) OR ((weight*) N2 (manag*)) OR ((well OR under) N1 nourish*) OR ((well OR under) N1 nourish*)))) **AND** (((regulation* OR government* OR law OR laws OR policy OR policies OR ((weight*) N2 (manag*)) OR ((information*) N2 (consumer-health* OR disseminat* OR literac* OR seek*)) OR ((diet OR nutrition OR sugar* OR fat OR fats*) N2 (restrict* OR act OR acts OR price OR pricing OR tax OR taxes OR taxation)) OR legal* OR illegal* OR hotline* OR quitline* OR (mass N1 (media OR medium OR communicat*)) OR ordinanc* OR prohibit* OR decree* OR enactment* OR mandator* OR campaign* OR ((pictorial OR graphic) N2 (warning*)) OR packaging OR mpower OR advertizing OR advertising OR legislat* OR statut* OR ((population-based OR public health) N2 strateg*)))) **AND** (((((digital*) N2 (devide* OR inequalit*)) OR socioeconomic* OR socio-economic* OR ((economic* OR educat* OR career* OR job OR jobs OR work OR profession* OR social* OR socio OR sociocultur* OR socio-cultur* OR sociodemograph* OR socio-demograph* OR occupat* OR employ*) N2 (status* OR achievement* OR condition* OR rank* OR rank* OR standing* OR state OR background* OR class* OR mobilit* OR deprivat* OR disadvantag* OR equalt* OR inequalt* OR low OR lower)) OR literac* OR illitera* OR ((abilit* OR capabilit* OR skill*) N2 (read* OR learn*)) OR ((living) N1 (standard*)) OR poverty OR income* OR employed OR unemployed OR remunerat* OR salary OR salaries))))

**Google Scholar (random top-200)**

diet|dieting|diets|dietary|"soft|sweet  drink|beverage"|"feed|eat|food|diet  behavior|behaviour|habit|preference|pattern"|"nutrition|food|vitamin|caloric|energy|vegetable|fruit|fat|sugar|carbohydrate|glucose|sodium|salt|cholesterol  intake|ingest" "government|policy|policies|"information health|dissemination|literacy|seeking"|"diet|fat|sugar ban|bans|banned|free|restriction" socioeconomic|"economic|education|social|socio|sociocultural| sociodemograph|occupation  status|achievement|poverty|income|employed|unemployed|salary|salaries
